# Arterial stiffness as a model to dissect chronic inflammation in FMF

**DOI:** 10.1186/1546-0096-13-S1-P93

**Published:** 2015-09-28

**Authors:** O Kukuy, A Leiba, L Mendel, A Benor, E Giat, O Perski, O Feld, Y Kessel, I Ben Zvi, M Lidar, A Livneh

**Affiliations:** 1 Sheba Medical Center, Heller Institute of Medical Research, Tel Hashomer, Israel; 2 Sheba Medical Center, Institute of Nephrology and Hypertension, Tel Hashomer, Israel; 3 Tel Aviv University, Tel Aviv, Israel; 4 Mount Auburn Hospital, Harvard Medical School, Department of Internal Medicine, Cambridge, MA, USA; 5 Omnistat Statistical Consulting, Tel Aviv, Israel; 6 Sackler School of Medicine, University of California at Berkeley, NY State program B.A. Molecular and Cell Biology, Berkeley, CA, USA; 7 Sheba Medical Center, Rheumatology Unit, Tel Hashomer, Israel; 8 Sheba Medical Center, Department of Internal Medicine F, Tel Hashomer, Israel

## Background

Familial Mediterranean fever (FMF) is an autoinflammatory disorder, characterized by short attacks of sterile serositis, successfully suppressed by continuous colchicine treatment. Subclinical chronic inflammation however, is a frequent finding in FMF, manifested with elevated levels of inflammatory markers

(CRP, SAA). While chronic inflammation is considered an important cardiovascular (CV) risk factor in most inflammatory disorders, findings on the impact of chronic inflammation in FMF are conflicting. Chronic inflammation is an important cause of arterial stiffness, an early sign and independent risk factor of cardiovascular atherosclerosis. Pulse wave velocity (PWV) measurement is a useful marker of arterial stiffness.

## Objectives

To study arterial stiffness in FMF and evaluate its predisposing factors.

## Methods

Eighty consecutive FMF patients without known CV risk factors, were enrolled to the study, during their scheduled visit to the clinic. Demographic, genetic, clinical, and laboratory data were obtained from their file and examination. Arterial stiffness was determined through pulse wave velocity (PWV), measured by Sphygmocor system algorithm (AtCor Medical, Sydney, Australia). The recorded PWV values were standardized per age and blood pressure and coded as PWV_Z, relating to the reference general population.

## Results

Compared with the adjusted general population, FMF patients displayed normal PWV values, even with a significantly reduced dispersion around the mean (5% vs 10%, p=0.02). In a trial to identify pro- or counteracting driving factors no statistically significant variables were found, yet the dose of the colchicine demonstrated the strongest trend for normalizing PWV.

## Conclusion

Arterial stiffness is not increased in FMF. Our data support a role for colchicine prophylaxis in this finding.

